# Instrumenting Beliefs in Threshold Public Goods

**DOI:** 10.1371/journal.pone.0147043

**Published:** 2016-02-09

**Authors:** Angela C. M. de Oliveira, John M. Spraggon, Matthew J. Denny

**Affiliations:** 1 Department of Resource Economics, 80 Campus Center Way, University of Massachusetts Amherst, Amherst, MA, 01003, United States of America; 2 Department of Political Science, Pennsylvania State University, University Park, PA, 16801, United States of America; Tianjin University of Technology, CHINA

## Abstract

Understanding the causal impact of beliefs on contributions in Threshold Public Goods (TPGs) is particularly important since the social optimum can be supported as a Nash Equilibrium and best-response contributions are a function of beliefs. Unfortunately, investigations of the impact of beliefs on behavior are plagued with endogeneity concerns. We create a set of instruments by cleanly and exogenously manipulating beliefs without deception. Tests indicate that the instruments are valid and relevant. Perhaps surprisingly, we fail to find evidence that beliefs are endogenous in either the one-shot or repeated-decision settings. TPG allocations are determined by a base contribution and beliefs in a one shot-setting. In the repeated-decision environment, once we instrument for first-round allocations, we find that second-round allocations are driven equally by beliefs and history. Moreover, we find that failing to instrument prior decisions overstates their importance.

## Introduction

One major obstacle to, and shortcoming of, the study of the relationship between beliefs and behavior is the potential for beliefs to be endogenous. As one example of many, consider the false-consensus effect. While the traditional assumption is that beliefs are exogenous and thus causally related to behavior, if individuals believe that others are ‘like them’ then endogeneity becomes a concern (e.g., [1]). With reciprocal or conditional-cooperator classes of preferences, the role of beliefs is fundamentally important to understanding individual decisions (e.g., [2, 3] and the review in [4]). If beliefs are endogenous, however, inferences based on the relationship between elicited beliefs and contributions are more difficult to interpret as the causal relationship is unclear. While an endogenous relationship between beliefs and behavior has been speculated, the presence of endogeneity remains an open empirical question. We thus design instruments, in an experimental setting, by providing information on the contributions of subjects who played the game previously. The mean and standard deviation of the information we provide act as valid and relevant instruments for beliefs allowing us to test for endogeneity. Perhaps surprisingly, we fail to find evidence that beliefs are endogenous in either the one-shot or repeated-decision settings. These results contribute to a growing discussion regarding the causal effect of beliefs in experimental games (see e.g., [5] for trust games and [6] for public good games).

Threshold Public Goods (TPGs) are a type of nonlinear public good [7]. For voluntary provision to occur, contributions must be large enough to cover the cost of the project. If contributions do not reach this threshold, then the good is not provided. In the TPG environment, the threshold (or provision point) introduces an infinite number of Nash equilibria to the game: a non-efficient, zero contribution Nash equilibrium exists, as do a multitude of efficient Nash equilibria where the threshold is exactly met. In comparison, the standard linear public good experiment has a unique Nash equilibrium at zero contributions. As a result, beliefs are particularly relevant in the threshold setting, both because of social preferences and because they help determine a Nash player’s best response.

Much of the previous research on TPGs has examined the impact of institutional design on efficiency. Returning contributions in the case that the threshold is not attained (a money-back guarantee, [7, 8]), refunding or re-investing contributions in excess of the threshold [9, 10] have both been shown to positively impact contributions and provision. These design features act to mitigate the risk associated with contributing, thus dampening the relationship between beliefs about others’ contributions and behavior. We therefore do not include opportunities for either rebates or refunds in our design.

The role of beliefs in TPGs has been almost completely ignored in the previous literature, even though beliefs are fundamental to understanding contributions in this decision environment. To the authors’ knowledge, the closest papers investigating beliefs examine donation matching [11] or step-level public goods (SLPG), which is a situation where individuals simply choose whether or not to contribute all of their endowment [12, 13]. If enough members of the group contribute, then the threshold is attained. Offerman, Sonnemans and Schram [12] establish that beliefs about the probability of contributing can be reasonably elicited and that beliefs weakly determine behavior. They also examine the process of forming expectation in SLPG games [13], comparing the evolution of beliefs between partners, strangers, and nature conditions. In this SLPG environment, elicited beliefs measure the subjective probability about the number of contributors. They find that beliefs are related to behavior: when a subject believes that their contribution will push the group over the threshold from non-provision to provision they are more likely to contribute. Since their focus is on the process of forming beliefs, they do not manipulate beliefs, or systematically examine the relationship between beliefs and contributions, which we do.

We conduct a TPG experiment on Amazon Mechanical Turk (AMT). In the baseline, we simply elicit beliefs. Previous research indicates that the belief elicitation procedure can impact stated beliefs [14, 15] and the very act of eliciting beliefs can alter behavior [16, 17]. However, players with asymmetric profit opportunities, where the immediate reaction and the strategic reaction differ, are the most likely to be impacted by the process of eliciting beliefs [18]. We do not test alternate elicitation procedures, our procedure is constant across treatments. Specifically, subjects are asked to state their belief about the contribution of the other three group members’ contributions as in [16]. In the first treatment, we manipulate beliefs without deception by providing them with information about previous contributions. The implications of providing information, which can alter beliefs about others actions or the social norm, has been demonstrated in several contexts, including charitable fund-raising [19–21] and public goods games [22]. We utilize a comparable methodology of informing subjects of actual decisions of previous subjects in order to manipulate their beliefs without deception. The information we provide consists of a set of ten decisions selected from those made by participants in earlier sessions. We use the mean and standard deviation of this information set of (over-identifying) instruments for beliefs, allowing us to both test for the validity of our instruments and to estimate the causal relationship between beliefs and behavior. We expect the mean of the information set to inform the expected average contribution, while the standard deviation should impact the perceived likelihood of actual contributions being close to the expected average. With less precise information, an individual’s degree of optimism (pessimism) would impact the elicited belief positively (negatively), which provides a channel through which the standard deviation could influence the elicited belief (we provide tests to confirm that it does). Econometric testing indicates that these instruments are relevant and valid. In the second treatment, subjects make two decisions—first without information, and then with information on previous contributions. This allows us to address the impact of history on decision making.

Perhaps surprisingly, and contrary to the concern in the profession, we fail to find evidence that in either a one-shot or repeated-decision environment that beliefs are endogenous using this design and elicitation. In contrast, history (lagged contribution decisions) does require instrumenting. We find that TPG contributions are driven equally by beliefs and prior contributions.

To the authors’ knowledge, there are only two similar papers in the public goods literature, both of which use a linear public goods setting [6, 23]. Bechtel and Scheve [23] conduct a multi-country field experiment, using an experimentally designed instrument and survey, to examine conditional cooperation and the causal effect of expected cooperation in a linear public goods game. They find a strong positive relationship between beliefs and contributions. They only have one instrument and do not discuss the necessity of instrumenting in their setting.

Smith [6] uses lagged contributions as instruments in a repeated linear public goods game. Other differences include the type of public good, subject pool, number of decisions, and level of feedback. He finds significant evidence of endogeneity of beliefs and that beliefs about others contributions are the primary determinant of own contributions. There are several differences between our study and Smith’s [6], besides the type of public good, which may impact the potential endogeneity of beliefs. Primarily, we elicit beliefs before the contribution decision whereas his belief elicitation is on the same screen as the contribution decision, which may enhance consistency between the two.

In other decision environments, several research teams have incorporated instruments into their experimental designs. For example, the causal effect of beliefs in the trust game are examined [5]. In this paper, they introduce random variation in the trustee response, while we manipulate the information set. They find that the OLS and IV estimates are not significantly different from each other, suggesting that beliefs are not endogenous in their setting. Note that their marginal effect on belief ranges from 0.54 to 0.56 which is in line with Smith’s IV estimates (ranging from 0.45 to 0.57 depending on the model) and ours (0.45 for the repeated-decision), suggesting that the relationship between beliefs and behavior may be relatively robust even across very different decision environments. This paper is unable to reject the exogeneity of beliefs in their prisoner’s dilemma environment. Additionally, democratic institution choice [24] and examine cash balances in auctions [25] are also examined.

Primarily our contribution is to the study of the relationship between beliefs and behavior. We investigate a TPG experiment, where we might expect beliefs to be more important than they are in a more standard linear public good experiment [13]. We propose instruments for beliefs and offer one of the first studies to systematically examine beliefs in this environment. We show that although beliefs are important to the decision making process, they are not endogenous in this setting. These results add to a broader literature which seeks to improve our understanding of how individuals interact when faced with social dilemmas [26–29]. We next detail the experimental design and implementation before turning to the results.

## Methods

### Experimental Design

We use the AMT platform to conduct a standard, single shot threshold public good game. AMT is an online labor market that has recently drawn increased interest as platform for conducting behavioral experiments. The platform has been examined in a number of recent methodological papers. Amir et al. [30] find relative parity between lab and AMT experiments, even when the stakes are very small in the online setting. Berinsky et al. [31] find that AMT subjects tend to be more attentive than undergraduate subjects or professionally recruited subjects in standard economic games and surveys. Horton, Rand and Zeckhauser [32] conduct a number of standard economic games on AMT and find that their results are consistent with results obtained in laboratory experiments. Goodman et al. [33] suggest implementing a quiz to check for the attentiveness of subjects and limiting of samples to the United States to prevent language problems (both of which we do here). The emerging consensus in the literature is that the results of studies conducted on the AMT platform are comparable to laboratory results for a number of standard games when care is taken with experimental design.

Potential subjects chose to participate in our experiment by selecting it from a list of thousands of Human Intelligence Tasks (HITs) on the AMT website. They were shown a recruitment screen with a link to the experiment website and conditions for participating. We limit the subject pool to English speaking subjects over the age of 18 living in the United States. We further implemented a program to check the subject’s unique AMT numerical identifier against previous participants in order to prevent any subject from participating more than once.

Once a potential subject chose to accept our task they were directed to an informed consent page. This informed consent page was approved, along with our study by the Institutional Review Board at the University of Massachusetts. Subjects were given information about what would be expected of them during the experiment and told they would earn 10 cents with an opportunity to earn a substantial bonus. We use this language in keeping with the norms of the AMT payment system. The 10 cents HIT served as the show-up fee, and was paid immediately, while the bonus consisted of the experimental earnings. All of our experiment web pages were viewed by subjects as a frame in the AMT website so subjects did not ever have to leave the AMT website. Subjects were paid within 48 hours, which is consistent with AMT best practices.

After reading and agreeing to the informed consent page, subjects were shown a set of instructions and examples, a sample of which is available in the Online Appendix (http://bit.ly/1MR7o96). The experimental framework is a four-player, one-shot TPG, with no refunds or rebates (thus maximizing the risk to cooperation, see [34] for a review). Payoffs, *π*_*i*_ to player *i* in a TPG game are as follows:

$$\pi_{i}=\left\{\begin{array}{ccc} E_{i}-c_{i} & \text{if} & \sum_{i}c_{i}<\text{PP}\\ E_{i}-c_{i}+R & \text{if} & \sum_{i}c_{i}\geq \text{PP} \end{array}\right.$$ (1)

where PP is the provision point, *E*_*i*_ is *i*’s endowment, *c*_*i*_ is *i*’s voluntary contribution and *R* is per-person the return from the TPG. Payoffs were determined according to Eq 1, where *E* = 10 cents is their endowment, *R* = 20 cents is the per-person value of the public good and PP = 20 cents is the provision point. Subjects could choose to contribute any integer between zero and ten, inclusive, to the group account. The remaining tokens are then allocated to their private account.

After completing the instructions, all subjects take a quiz to determine their understanding and to assist with eliminating computerized agents. Subjects in all treatments receive the same instructions and quiz. After the quiz, subjects continue on to complete either the No Information, Information, or History treatment of the experiment. In the No Information (NI) treatment, we elicit subject’s subjective beliefs about the average contribution of the other three members of their group (as in [16]). We offer a 20 cent bonus for guessing correctly. Paying for correctness has a few advantages in our setting. First, for the AMT subject pool we needed a mechanism that was easily and quickly explained. Second, it avoids many of the problems discussed in the literature [35]. In linear public goods games, eliciting beliefs before the allocation decision has been shown to reduce contributions and increase equilibrium play [17]. For our design, multiple equilibria exist, and so the impact of eliciting beliefs on equilibrium play is less clear. If the elicitation increases strategic thinking, then subjects may play closer to their best-response function than they otherwise would. This is not the research question we address here, and the elicitation is constant across all treatments. We have no a priori reasons to expect that subjects in separate treatments would react differently to the elicitation.

After the belief elicitation, subjects make their contribution decision, complete a brief survey, and exit the experiment. Upon completion, each subject is matched with three other subjects who have not yet been matched and paid their bonus. Specifically, and unknown to the participants, subjects were matched in order of completion. Along with the bonus, subjects received an accompanying message explaining how their bonus was calculated. The asynchronous nature of our experiments differs from previous Mechanical Turk studies where subjects are recruited to an online waiting room and then play synchronous games, but this difference is beneficial for several reasons. First, it does not allow for collusion because subjects are not aware of how they are being grouped. Second it allows for subjects to complete the experiment in a very short amount of time, thus maximizing the effective payoff to participation. If the total contribution to the group account equaled or exceeded 20, then each group member receives 20 cents plus however many cents they kept in their personal account.

In Information (I) treatment, subjects participate in the same one-shot TPG except that we exogenously manipulate beliefs after subjects complete the quiz and before we elicit their beliefs.

In order to provide a stronger test of the impact of the manipulated information on beliefs and behavior, we conduct the History (H) treatment. In this treatment, subjects make two allocation decisions (H-NI and H-I), and are paid for both. Note that the matching and payment does not occur until after the entire experiment is completed, so there is no feedback between decisions. For the first decision (H-NI), subjects first make a No Information allocation decision. After making this decision, subjects are then shown an information set, complete a second belief elicitation, and make a second allocation decision (H-I), as in the Information treatment.

### Implementation

Data for this study was collected through AMT. A total of 2039 subjects participated in Study 1 between July 2013 and June 2014. Once we implement our screening for automated players, we are left with a total of 1447 usable observations, 445 in the NI treatment, 564 in the I treatment and 438 in the H treatment. We implemented a quiz to gauge attentiveness, following the procedures in [33]. Subjects had to answer two multiple choice questions (with five possible answers). They were given hypothetical contributions and asked to calculate payoffs. In order to proceed with the experiment, subjects had to correctly answer both questions in the same attempt. We then discarded all observations where subjects failed the quiz more than four times. These subjects earned an average of 24.8 cents in the NI and I treatments and 50.7 cents in the H treatment, in addition to the 10 cents they were paid for accepting the task. The session lasted an average of 3 minutes and 40 seconds (4 minutes and 20 seconds) [4 minutes and 52 seconds] for the NI (I) [H] treatment. If a computer error made a subject’s data unusable, they were paid a $1 bonus and have been excluded from these numbers. A concern raised with Mechanical Turk is that the incentives are too low to impact behavior. Research suggests that subjects from Mechanical Turk respond to incentives in much the same way as other populations [32].

### Instruments

**Fig 1:**
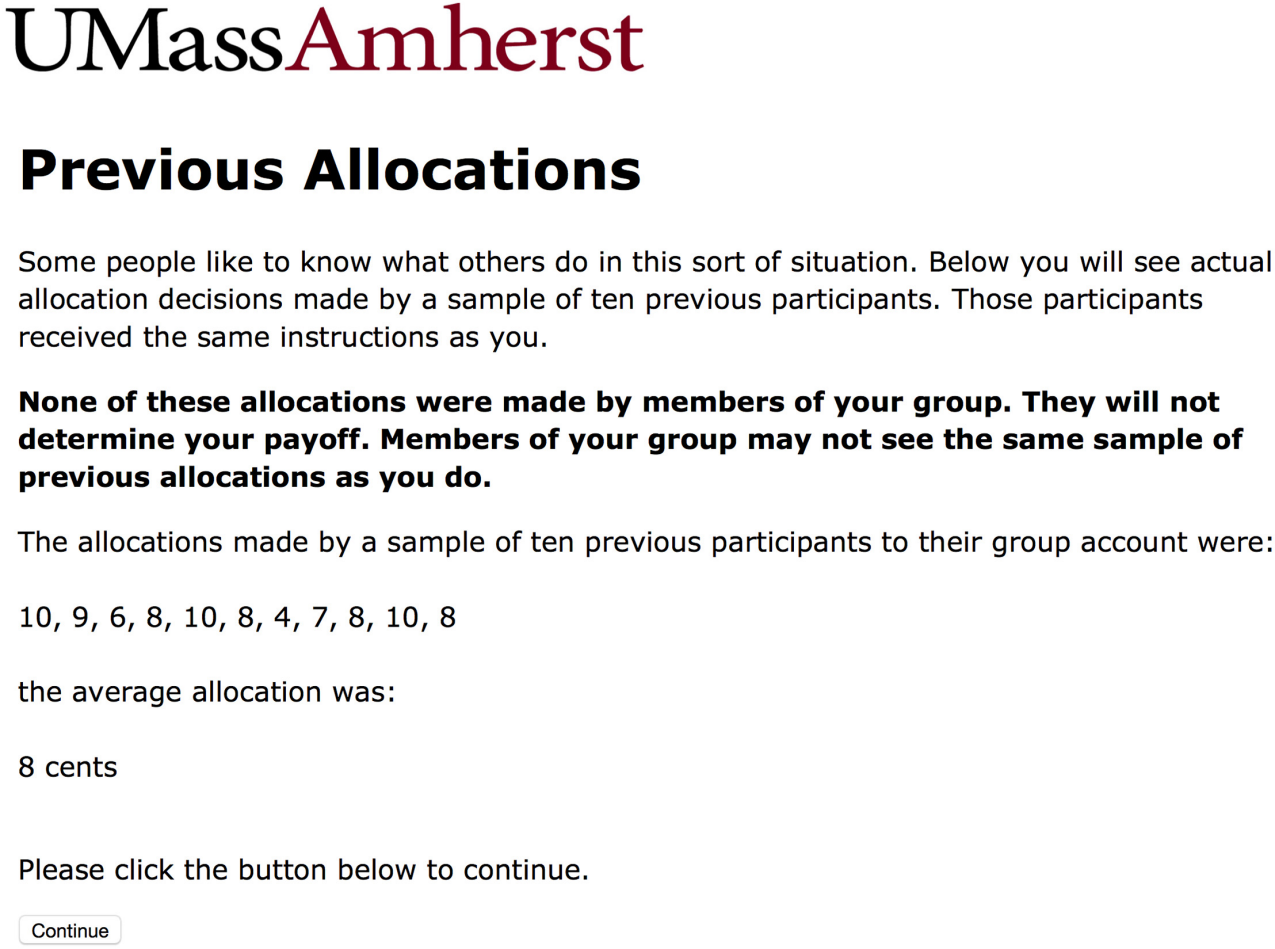
Sample Information Manipulation.

We create instruments by exogenously manipulating the beliefs of subjects in a manner similar to [21]. We show subjects an information set containing contributions (and the mean of those contributions) from ten anonymous, randomly selected, previous participants. Subjects are specifically (and truthfully) told that these previous participants will not be in their group and that the contributions of these previous participants will not impact their payoff. We therefore ensure that the instrument is exogenous and that any influence on contributions occurs through beliefs and not from strategic considerations. A sample manipulation is shown in Fig 1.

**Fig 2:**
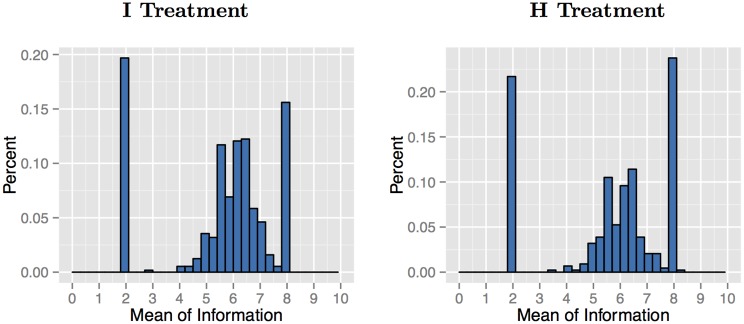
Means of Observed Information Sets. **Note:** The spikes at 2 and 8 are from the experimenter-chosen information sets.

**Fig 3:**
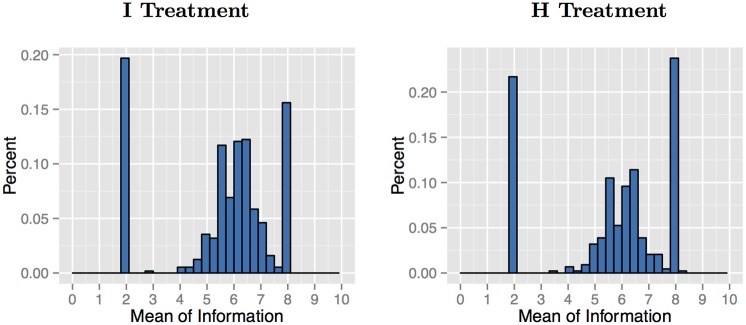
Standard Deviations of Observed Information Sets. **Note:** The spikes at 0.89 and 1.84 are from the experimenter-chosen information sets.

Next, we need to ensure that there is sufficient variation in the information sets. For both I and H, we allow the natural variation in information by randomly drawing previous contributions. For a subset of the sample (196 out of 564 in I and 199 out of 439 in H), we use a stronger manipulation of beliefs, where we select specific prior contributions to create four information sets, two with a low mean (2) and two with a high mean (8) (similar to the controlled previous donor information in [21]). For each mean, we also have a lower standard deviation (0.89) and a higher standard deviation (1.84). Participants randomly participate in one of the four combinations. Note that the low and high mean could both discourage contributions. As illustration, if an individual believed that individuals contributed 2 (8) each, for a total of 6 (24), then it would never be a money-maximizing best response to contribute. Within the each standard deviation, numbers were chosen to mirror each other. These controlled information sets are shown in Online Appendix Table 1 (available at http://bit.ly/1MR7o96). The distribution of the means and standard deviations are shown in Figs 2 and 3.

Since beliefs may be endogenous, one instrument is required to just-identify the model. However, if the model is just-identified, then we are unable to test whether instruments are valid. We therefore propose two instruments in order to over-identify the model. The instrumental variables (IVs) we propose are the mean and standard deviation of the observed information set. The mean of the information set is meant to inform the expected average contribution. Beliefs regarding expected contributions of others are important for TPGs since one’s optimal decision depends on the contributions of others.

The standard deviation captures the precision of the information treatment. With less precise information, an individual’s degree of optimism (pessimism) would impact the elicited belief positively (negatively). Thus, while the standard deviation of information does not have to influence beliefs, there is a channel through which it could influence beliefs. Other aspects of the information set may impact beliefs as well. However, using too many instruments can give rise to weak instrument issues (see e.g. [36]). We use the standard deviation as an over-identifying instrument so that we can test for the validity of the instruments. Estimates without using the standard deviation are not significantly different (available upon request).

In order to consider the mean and standard deviation of the information set as proposed instruments, first we need to confirm the first-order condition, that the instruments are valid (uncorrelated with allocations after controlling for the other determinants of behavior). Next, to satisfy the second-order condition, the manipulation has to actually ‘work’: that is, the proposed instruments need to be relevant (correlated with beliefs).

For our preliminary evaluation of the validity of the proposed instruments, we need to confirm that they do not significantly, independently influence contributions once the other explanatory variables have been included in the regression. That is, controlling for beliefs, the mean and standard deviation of the information set do not offer additional explanatory power. If either of the proposed instruments were significant in this regression, it would indicate that they would be correlated with the errors from a regression of beliefs on allocations, and thus not be considered valid.

Results from the OLS regression are shown in Table 1. The dependent variable is the amount allocated to the group account. Marginal effects are shown, with standard errors in parentheses. We see that both of the proposed instruments are statistically and economically insignificant. The instruments thus meet the first criteria. Both instruments are also statistically insignificant when regressed independently (available on request).

**Table 1:** Validity: Do Proposed Instruments Independently Impact Contributions?

	**I Treatment**
Belief	0.713*** (0.058)
Information Mean	-0.026 (0.044)
Information Standard Deviation	0.028 (0.116)
Constant	1.977*** (0.356)
N	564
Adjusted *R*^2^	0.2361

***p ≤ 0.001

*Notes:* OLS regressions, with the allocation to the TPG as the dependent variable. The regression includes the proposed instruments after accounting for the other relevant variables in the regression. Marginal effects are shown, with standard errors in parentheses.

**Fig 4:**
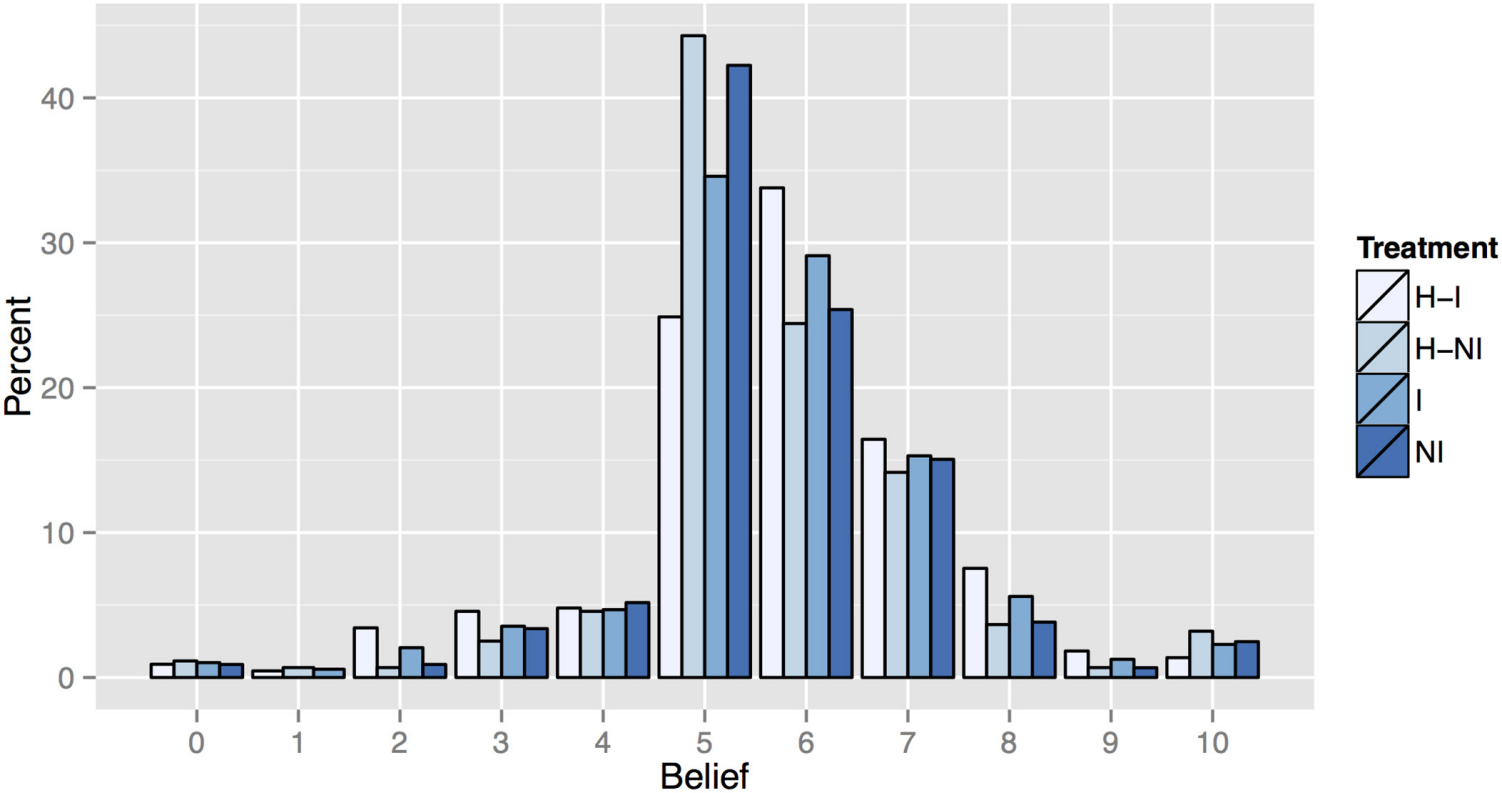
Beliefs of Individual Allocations to the Group Account, by Treatment.

We next need to consider the relevance of the proposed instruments. We address relevance in two manners. First, we need to confirm that providing information impacts beliefs. Fig 4 shows the distribution of beliefs for each of the four decisions (NI, I, and both H decisions). There is a significant impact of providing any information on the distribution of beliefs (Kolmogorov-Smirnov, all *p* < 0.01). We see a shift in the modal response of 5 in the decisions with no information (NI and H-NI) to 6 in the decisions where information was received (I and H-I, proportions tests all *p* < 0.01).

For the mean and standard deviation of the information set to be relevant instruments, they need to be significantly correlated with beliefs. Table 2 provides the first-stage regression, the OLS estimates for the relationship between the proposed instruments and the elicited beliefs for the I treatment. We focus on the I treatment for evaluating the potential of these instruments because the H-I treatment will also have to account for history, which is endogenous by construction. We will return to the analysis of H-I in the Results Section. There is a strong positive relationship between the mean of the information set and the elicited belief. Likewise, there is a positive relationship between the standard deviation of the information set and the elicited belief. Thus, both the mean and standard deviation of the information set are strongly correlated with beliefs, suggesting that they are relevant. We will provide further tests of the quality of the proposed instruments along with the econometric results in the Results Section.

**Table 2:** Relevance: Are Potential Instruments Correlated with Beliefs?

	**I Treatment**
Information Mean	0.281*** (0.029)
Information Standard Deviation	0.256** (0.084)
Constant	3.531*** (0.212)
N	564
Adjusted *R*^2^	0.1677

**p ≤ 0.01,

***p ≤ 0.001

*Notes:* The first-stage regression is modeled as OLS with the elicited belief as the dependent variable. Marginal effects are shown, with standard errors in parentheses.

## Results

**Fig 5:**
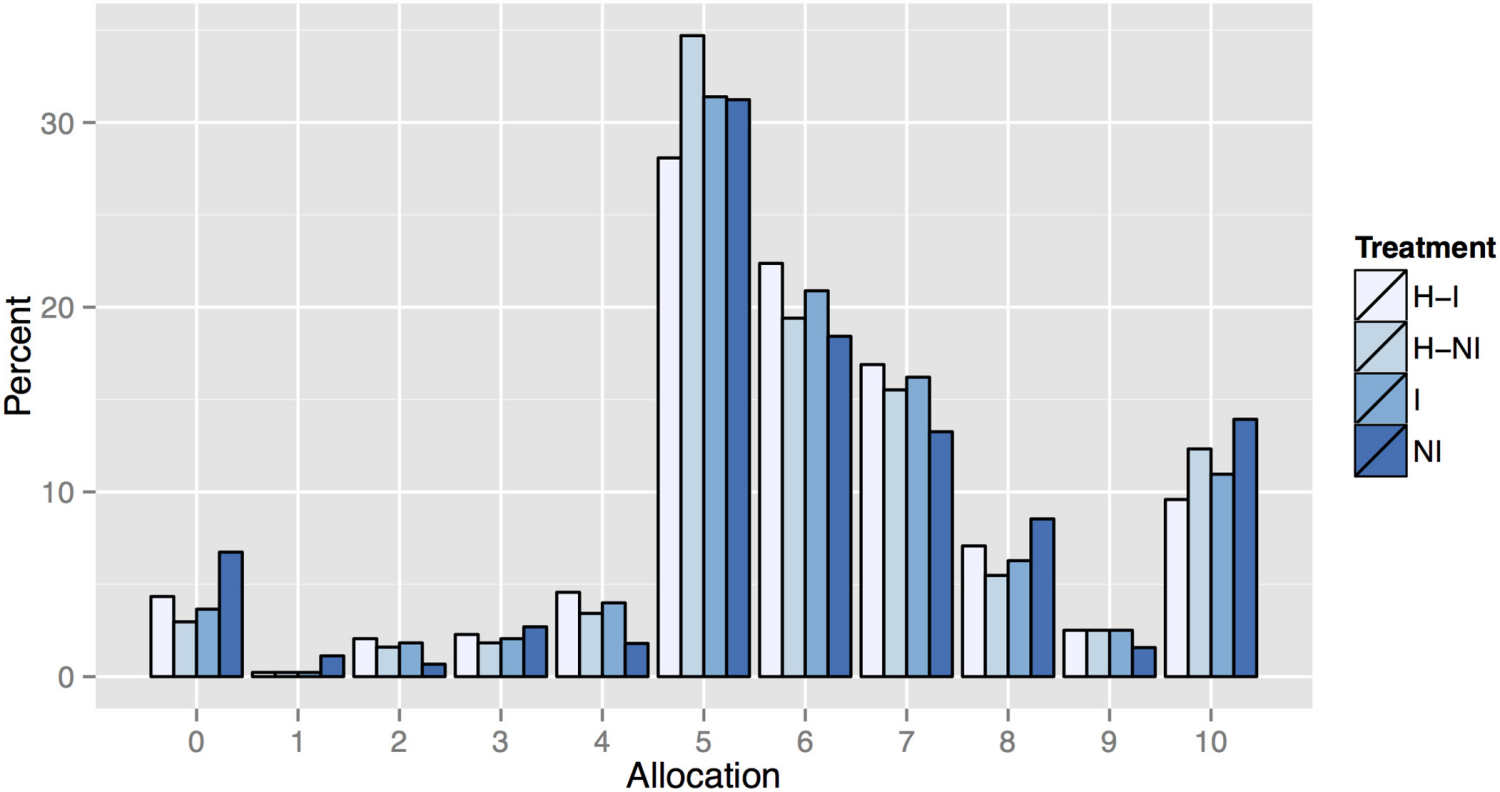
Individual Allocations to the Group Account, by Treatment.

Now that we have two potential instruments and evidence suggesting that they are valid and relevant, we turn to our analysis of the relationship between allocations and beliefs. Table 3 presents the average allocation, belief, and number of individual observations for each of our treatments and decisions. The distribution of beliefs by treatment was shown previously in Fig 4 and the distribution of allocations is shown below in Fig 5.

**Table 3:** Average Allocations and Elicited Beliefs, by Treatment.

*One-Shot*	NI	I
Allocation	5.99 (2.51)	5.86 (2.13)
Belief	5.63 (1.42)	5.57 (1.48)
N	445	564
*History*	H-NI	H-I
Allocation	6.11 (2.15)	5.97 (2.20)
Belief	5.62 (1.51)	5.73 (1.62)
N	438	438

Standard errors in parenthesis.

Since subjects do not interact or receive any feedback, we consider each individual to be an independent observation for this analysis. The first set of rows in Table 3 contains the data from the one-shot treatments, NI and I. The second set of rows contain data from the H treatment, where each subject makes two decisions with a surprise re-start and information manipulation after the first decision. H-NI is in the first column of data and H-I is in the second. Thus, a vertical comparison holds constant the information condition (with or without the information manipulation) while a horizontal comparison provides the impact of receiving any information.

In all treatments, allocations are significantly higher than beliefs (*t*-tests, all *p* < 0.01). We do not see treatment differences in the average allocation to the group account (*t*-tests, all *p* > 0.05). Regarding the distribution of allocations to the group account, there are no significant differences between treatments. The only exception is that the distribution of allocations is significantly different within the H treatment, with the first decision (H-NI) having a different distribution than the second (H-I, Kolmogorov-Smirnov, *p* < 0.01).

We next turn to our analysis of the relationship between allocations and beliefs in the one-shot decision, provided in Table 4. The first column shows the estimates without IVs (using OLS) and the second column shows the estimates with IVs (using 2SLS). While one might expect a nonlinear relationship between beliefs and contributions (based on the best response functions), testing indicates that a linear specification is an approximate description of the relationship (results available upon request).

**Table 4:** Relationship between Allocations and Beliefs in a One-Shot TPG: with and without Instruments.

	**I Treatment**	
	No IV	IV
Belief	0.702*** (0.053)	0.648*** (0.128)
Constant	1.948*** (0.304)	2.247** (0.715)
N	564	564
Adjusted *R*^2^/Centered *R*^2^	0.2383	0.2382
*Tests:*		
Anderson LM (*p*)	96.232 (0.00)	
Cragg-Donald Wald *F*	57.706	
Sargan Stat (*p*)	0.161 (0.69)	
Wu-Hausman *F* (*p*)	0.213 (0.64)	
Durbin-Wu-Hausman *χ*^2^ (*p*)	0.214 (0.64)	

**p ≤ 0.01,

***p ≤ 0.001

*Notes:* The No IV specifications are modeled as OLS. The IV specifications are modeled using 2SLS. The instruments used are the mean and standard deviation of the information set. Marginal effects are shown, with standard errors in parentheses.

Tests for the quality of and need for instruments are at the bottom of the table. The Anderson LM test indicates that we have full column rank in all cases. This is a test of under-identification, and the p-value of zero indicates that both instruments are relevant. Next is the Cragg-Donald Wald *F*, which is a test of weak identification. If the instruments were correlated with the potentially endogenous regressor but only weakly, then the estimator would not perform well. The test statistic is 57.7, which is above the Stock-Yogo 10% critical value of 19.93, indicating that our proposed instruments are not weak (Stock and Yogo [37]). Finally, the Sargan-Hansen test evaluates whether the instruments are jointly valid (uncorrelated with the error). The *p*-value of 0.69 for the Sargan Statistic indicates that we fail to reject validity. Combined with the evidence from Tables 1 and 2 above, we thus conclude that the proposed instruments are both valid and relevant in this decision framework.

We find a surprising result: despite the serious concerns about belief endogeneity in the profession, we do not find that beliefs are endogenous in this setting. The *p*-value of 0.64 for both the Wu-Hausman and Durbin-Wu-Hausman tests indicate that both estimators are consistent. If beliefs were endogenous, then OLS would be inconsistent. Thus, we fail to reject the exogeneity of beliefs. In the one-shot setting (I Treatment) contributions are made up of a base contribution of approximately 2 cents plus 70% of the elicited belief.

To examine the robustness of this result, we next turn to the H Treatment, where individuals make two decisions, with a surprise re-start and receive the information manipulation in between the decisions. In order to examine the second decision (H-I), we also need to include the first-round decision to avoid omitted variable bias. However, the first-round decision (H-NI) is essentially a lagged variable, thus we will need to include another instrument to account for this uncontrolled individual heterogeneity. Specifically, we need something that will influence the first-round decision directly but will not directly influence the second round decision. Given the observed result for the I treatment, we test first round beliefs as a potential instrument for 1^st^ round allocations. Results (without and with IVs) are shown below in Table 5.

**Table 5:** Relationship between Allocations, Beliefs and History: with and without Instruments.

	**H-I**	
	No IV	IV
Belief	0.442*** (0.043)	0.453*** (0.082)
1^st^ Allocation (H-NI)	0.624*** (0.033)	0.498*** (0.076)
Constant	-0.372 (0.266)	0.335 (0.414)
N	438	438
Adjusted *R*^2^/Centered *R*^2^	0.6117	0.5989
*Tests:*	
Anderson LM (*p*)	77.856 (0.00)	
Cragg-Donald Wald *F*	31.274	
Sargan Stat (*p*)	0.051 (0.82)	
*Beliefs:*
Wu-Hausman *F* (*p*)	0.781 (0.38)	
Durbin-Wu-Hausman *χ*^2^ (*p*)	0.786 (0.38)	
*1^st^ Allocation:*
Wu-Hausman *F* (*p*)	4.744 (0.03)	
Durbin-Wu-Hausman *χ*^2^ (*p*)	4.736 (0.03)	

***p ≤ 0.001

*Notes:* The No IV specifications are modeled as OLS. The IV specifications are modeled using 2SLS. The dependent variable is the amount allocated to the group account in the second decision of the H treatment (H-I). Instruments used are the mean and standard deviation of the information set as well as the elicited belief from the first allocation decision. Marginal effects are shown, with standard errors in parentheses.

As with the one-shot setting, the Anderson LM and Cragg-Donald tests indicate that the instruments are relevant and the Sargan Statistic indicates that the instruments are jointly valid. The *p*-value of 0.38 for both the Wu-Hausman and Durbin-Wu-Hausman tests of the Belief variable indicate that both estimators are consistent. Therefore, even in our repeated-decision environment we fail to reject the exogeneity of beliefs. The *p*-value of 0.03 for both the Wu-Hausman and Durbin-Wu-Hausman tests of the 1^st^ Allocation (H-NI) indicate that OLS is inconsistent and that the 1^st^ Allocation needs to be instrumented. Once accounting for an individual’s first decision, which we refer to as their history, and instrumenting for potential endogeneity, we see that contribution behavior is equally driven by the earlier decision and beliefs (*χ*^2^(1) = 0.10, *Pr* > *χ*^2^ = 0.75). The lagged variable continues to need IV methods applied, and failure to do so would have over-stated the importance of history.

## Discussion

We investigate the oft-held belief that experimentally elicited beliefs are endogenous using a TPG decision environment. We create instruments by exogenously manipulating subject’s beliefs in a single shot setting where there is no opportunity for endogenous belief formation. By showing subjects a list of 10 previous contributions and their mean we make this information easy to understand and encapsulate other important aspects of the implied distribution over contributions such as its variance.

We find that our proposed instruments, the mean and standard deviation of the information set, are valid and relevant instruments. Perhaps surprisingly, we fail to reject the exogeneity of elicited beliefs in either our one-shot or repeated-decision settings. TPG allocations are determined by a base contribution and beliefs in a one shot-setting. In the repeated-decision environment, once we instrument for first-round allocations, we find that second-round allocations are driven equally by beliefs and history. Failing to instrument history overstates its importance. Interestingly, our results are in line with previous research in that our estimate of the effect of beliefs on decision making is comparable to Smith’s [6] estimate of beliefs on decision making in a linear public good experiment.

Future research should examine the relationship between the belief elicitation procedure and potential endogeneity of beliefs. With appropriately designed IVs, we can test the influence of belief elicitation methodology on the potential endogeneity of beliefs. Our elicitation was very specific: beliefs about the other three players, paying for exact correctness, and beliefs were elicited before the decision. Any aspect of these design features could contribute to our failure to reject the exogeneity of stage-one beliefs. Additional careful research will be required to confirm whether the result holds generally, or whether some elicitation procedures are ‘cleaner’ than others for this purpose.
